# Coupled DFT and SCAPS-1D investigation of a novel small-molecule for organic solar cells

**DOI:** 10.1039/d5ra04889c

**Published:** 2025-08-26

**Authors:** Mustafa K. A. Mohammed, Asmaa Yahya Al-Baitai, Saifaldeen M. Abdalhadi, Vicky Jain, Asha Rajiv, Mayank Kundlas, Helen Merina Albert

**Affiliations:** a College of Remote Sensing and Geophysics, Al-Karkh University of Science Baghdad 10011 Iraq dr.mustafa@kus.edu.iq; b College of Science, University of Warith Al-Anbiyaa 56001 Karbala Iraq; c Chemistry Department, College of Science, Al-Nahrain University Baghdad 10011 Iraq; d Marwadi University Research Center, Department of Chemistry, Faculty of Science, Marwadi University Rajkot Gujarat India; e Department of Physics & Electronics, School of Sciences, JAIN (Deemed to be University) Bangalore Karnataka India; f Centre for Research Impact & Outcome, Chitkara University Institute of Engineering and Technology, Chitkara University Rajpura 140401 Punjab India; g Department of Physics, Sathyabama Institute of Science and Technology Chennai Tamil Nadu India

## Abstract

Herein, we theoretically developed and studied a novel small-molecule donor, SA1, a phenazine-based derivative, for organic solar cells (OSCs). Density functional theory (DFT) analysis was performed to optimize its electronic and molecular characteristics, which were then employed as input parameters for solar cell capacitance simulator (SCAPS-1D) calculations to explore the photovoltaic properties of the OSC. The SA1 structure incorporated a thieno[3,2-*b*]thiophene bonded with thioxothiazolidin-4-one as an electron-rich part to improve the lowest unoccupied molecular orbital (LUMO) level, while a dibenzo[*a*,*c*]phenazine core operated as a weak electron-acceptor part to enhance the highest occupied molecular orbital (HOMO) level. The influences of different layer parameters, such as thickness, doping level, bulk defect density, and density of states, were systematically studied. The effects of fundamental merits, such as electrode work function, parasitic resistances, and temperature, on OSC efficiency were also discussed. After optimization, our OSC device demonstrated a remarkable power conversion efficiency (PCE) of 16.94%, a fill factor (FF) of 87.34%, an open-circuit voltage (*V*_OC_) of 1.27 V, and a short-circuit current (*J*_SC_) of 15.26 mA cm^−2^. Additionally, the designed SA1-based cell showed predictable high thermal stability at 145 °C based on simulation results, along with a high external quantum efficiency (EQE) of 95% in the visible spectrum.

## Abstract 2

Herein, we theoretically developed and studied a novel small-molecule donor, SA1, a phenazine-based derivative, for organic solar cells (OSCs).

## Graphical abstract

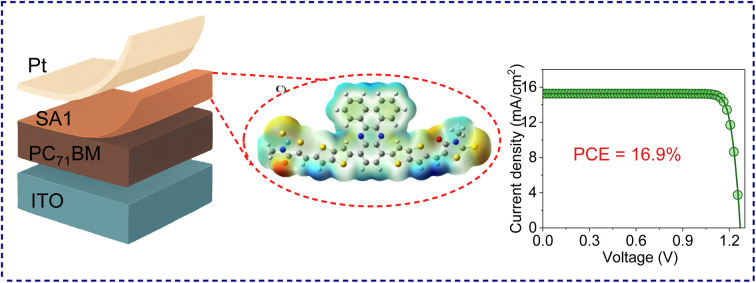

## 1. Introduction

The global demand for electricity is increasing exponentially, driven by population growth and technological advancement. Fossil fuels, which account for around 80% of the energy supply, are limited in capacity.^1^ As a potential alternative to fossil fuels, solar cells have attracted considerable interest in recent decades because of the important issues of global warming, depletion of natural resources, and environmental concerns.^2,3^ Organic solar cells (OSCs) have the potential to play a key role in the future power infrastructure.^4,5^ OSCs have demonstrated rapid progress based on bulk heterojunction or layer-by-layer structures *via* utilizing well-matched polymer donors and narrow bandgap (*E*_g_) acceptors.^6,7^ In the OSC architecture, the donor and acceptor charge transport materials form a solid-state blend, promoting exciton dissociation at the donor–acceptor interface and enabling simultaneous transfer of holes and electrons to their respective electrodes.^8^ The optimization of interfaces between layers is critical for enhancing charge extraction and minimizing recombination losses in solar cells, as demonstrated in recent studies.^9–11^ It is important to know the energy level requirements for donor molecules, elucidating how suitable energy levels minimize exciton binding energy. Moreover, donor molecules must promote efficient charge transfer with low energy losses.^12^ The molecular structure should ideally minimize the electronic coupling coefficient (*κ*) and energy difference between the HOMO and LUMO (Δ*E*) to enhance efficacy in light-harvesting or charge transfer. A side chain or peripheral group is necessary to bestow solubility, enabling the processing and fabrication of donor materials into thin films while maintaining fused-ring cores for π-conjugation and rigidity.^13,14^

Small molecules (SMs) have emerged as competitive alternatives to conjugated polymers in OSCs, owing to their distinct merits, such as well-defined chemical structures, reduced batch-to-batch variation, and easier purification.^15^ A class of SMs that has garnered significant attention for use as donor material in OSCs is heterocyclic nitrogen-containing compounds. This class includes versatile compounds with diverse chemical structures, such as phenothiazine,^16^ phenoxazine,^17^ carbazole,^18^ pyrrole,^19^ and quinone.^20^ Among these candidates, phenazine derivative compounds consisting of two fused aromatic rings with one or two nitrogen heteroatoms have been extensively studied in OSCs and other optoelectronic devices.^21,22^ Khanam *et al.* used DFT computation to design non-fullerene SMs containing a phenazine core modified with a variety of substituents at the terminals. The designed molecules showed reduced *E*_g_ with enhanced absorptivity.^23^ Later, Li and co-workers incorporated a selenium (Se) additive into phenazine-based SMs for OSCs. The synergistic effect of phenazine cores and Se substitution increased light-harvesting capability, resulting in OSC devices with high *J*_SC_ and a PCE of 17.69%.^24^ Recently, Wang *et al.* synthesized different SMs based on a phenazine unit with an alkyl chain, π-bridges, and terminal halogenation for OSCs. Three molecules demonstrated near-IR absorption, appropriate energy levels, high stability, and good intermolecular stacking. Consequently, the champion OSCs achieved a performance of 10.11%.^25^

Extending these developments, our work intends to develop and evaluate a new phenazine-based small molecule for OSCs. A novel phenazine, coded as SA1 (((5*Z*,5′*E*)-5,5′-((5,5′-(dibenzo[*a*,*c*]phenazine-10,13-diyl)bis(thieno[3,2-*b*]thiophene-5,2-diyl))bis (methanylylidene))bis (3-ethyl-2-thioxothiazolidin-4-one))) was designed theoretically by combining a dibenzo[*a*,*c*]phenazine unit with thieno[3,2-*b*]thiophene and thioxothiazolidin-4-one groups in an SM donor. The DFT calculations were utilized to investigate the physical and chemical properties, which were subsequently used as input for SCAPS-1D simulations to predict the photovoltaic performance. The optimized OSC recorded a PCE of 16.94%, a FF of 87.34%, a *J*_SC_ of 15.26 mA cm^−2^, and a *V*_OC_ of 1.27 V. The remarkable photovoltaic performance achieved by the SA1-based OSC highlights a novel molecular structure that offers a promising direction for next-generation high-efficiency organic solar cells.

## 2. Methodology

### 2.1. Donor designing and computational studies

**Fig. 1:**
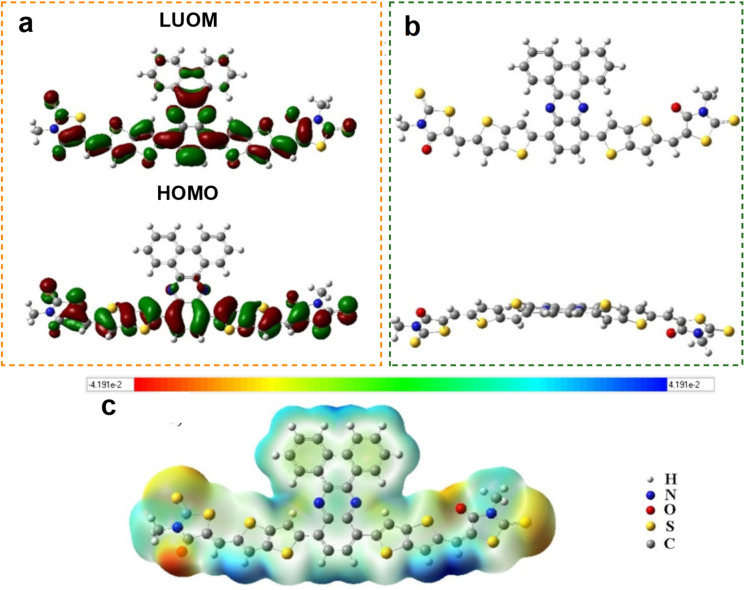
Description of the designed SA1 organic compound. (a) The electron distribution of the HOMO and LUMO. (b) The optimization structure. (c) The electrostatic potential analysis.

The SA1 compound was designed as a donor material in OSC, as shown in Fig. 1. The design idea of the SM was represented by using dibenzo[*a*,*c*]phenazine as a core in the center of the molecule with two arm molecules of thieno[3,2-*b*]thiophene and thioxothiazolidin-4-one bonded in the 5,5′ position of phenazine. The phenazine core was used due to the distinct advantages, such as enhanced photochemical stability, strong light absorption, and efficient charge transport mobility.^26^ The thioxothiazolidin-4-one and thieno[3,2-*b*]thiophene were used as a rich electron part to improve the LUMO, and the phenazine core was used as a weak acceptor part to enhance the HOMO of SA1.^27^ Gaussian 09W performed all computational studies for SA1. The geometry optimizations and frequency calculations were performed using DFT, followed by TD-DFT calculations to simulate electronic transitions, all using the Becke's three-parameter hybrid function combined with the Lee–Yang–Parr correlation function (B3LYP) functional and 6-311++G** basis set in vacuum. The simulated vibrational frequencies of SA1 have no imaginary peaks, indicating that the simulation has true energy level minima. Fig. 1a shows the electron distribution of HOMO and LUMO for SA1. The electron distribution in HOMO was delocalized in two arms of the compound with energy 5.67 eV, and the electron distribution of LUMO was delocalized in the center and arms of the compound with energy 3.82 eV, which gave a good position for the injection of the electron to the LUMO of the acceptor.

Fig. 1b illustrates the optimization structure for SA1. The structure exhibited a moderately planar backbone, as evidenced by DFT-calculated dihedral angles of 32.74° between the core and thieno[3,2-*b*]thiophene. Additionally, the small dihedral angle of 0.21° between the thieno[3,2-*b*]thiophene and terminal unit. This planarity facilitates π–π stacking in the solid state, enhancing intermolecular charge transport.^28^ Additionally, the extended conjugation from the core through the arm of the molecule enables efficient electronic delocalization, which contributes to the narrow optical bandgap and strong absorption in the visible region.^29^Fig. 1c shows a graphical representation of the electrostatic potential (ESP) generated by a molecule, allowing its charge distribution to be perceived more easily. The high electron cloud in the SA1 was represented in most of the molecule and depicted in green and blue colours. In contrast, a small area of red colours was located in the O atoms of thioxothiazolidin-4-one.^30^ Generally, the ESP results indicate that the SA1 compound behaves as a nucleophile, particularly in the two benzene rings of the phenazine and the thieno[3,2-*b*]thiophene moiety. The electron-rich (negative potential) regions located on the phenazine core and thieno[3,2-*b*]thiophene units suggest a strong tendency for π–π stacking, which is favorable for efficient hole mobility.

### 2.2. OSCs device simulation

The SCAPS-1D can be used to calculate energy bands, current–voltage characteristics (*J-V*), and the EQE spectrum by solving the Poisson and electron–hole continuity equations. This tool can also determine the distribution of electric fields (*E*) across layers and interlayers, in addition to recombination patterns. The following basic equations for carriers are used in this software:^31,32^1
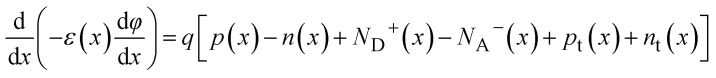
2
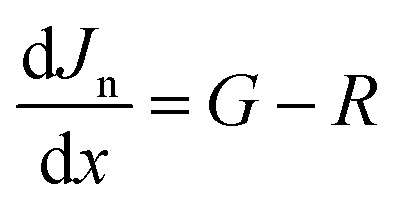
3
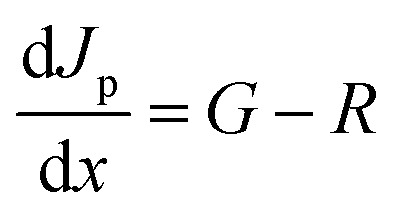


The charge carrier transport processes can be explained by the drift-diffusion approach:4
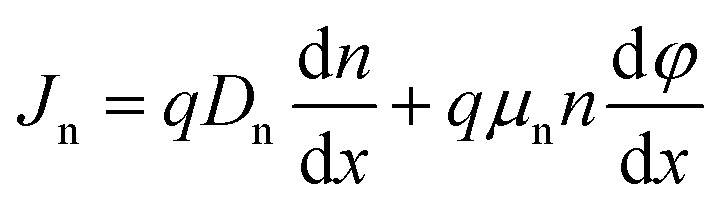
5
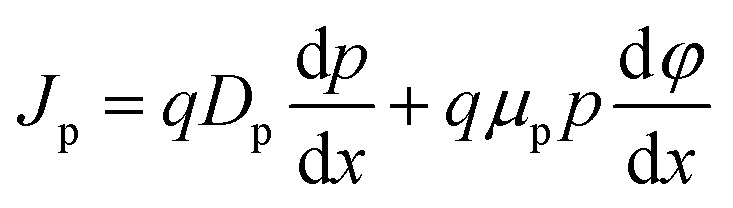
where *ε*(*x*): the position-dependent permittivity of the material, *ψ*: electrostatic potential, *q*: elementary charge, *n*(*x*) and *p*(*x*) are density of free electrons and holes, respectively. *N*_D_^+^ and *N*_A_^−^ are ionized donor and acceptor dopant concentrations. *n*_t_(*x*) and *p*_t_(*x*) are the density of trapped electrons and holes, respectively, *R*: recombination rate of carriers, *G*: generation rate of carriers, *J*_n_: electron current density, *J*_p_: hole current density, *D*_n_: diffusion coefficient for electron, *D*_p_: diffusion coefficient for hole, *μ*_n_: electron mobility, *μ*_p_: hole mobility.

**Fig. 2:**
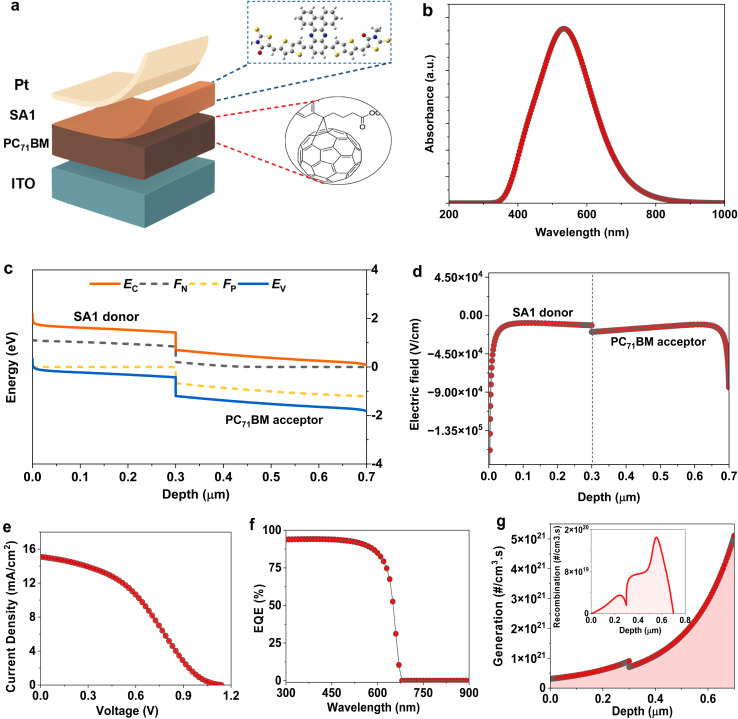
The architecture and optoelectronic properties of primary planar-heterojunction OSC device. (a) The device structure. (b) Absorption profile of SA1 compound. (c) Energy band diagram. (d) Electric field distribution. (e) The *J*–*V* curve of designed OSC. (f) The corresponding EQE profile. (g) Generation-recombination profile of simulated OSC.

All calculations are conducted at an atmospheric temperature of 300 K under the AM1.5 solar spectrum and a frequency of 1 MHz. Furthermore, the values of 2 Ω cm^2^ for series resistance (*R*_S_) and 10^4^ Ω cm^2^ for shunt resistance (*R*_Shunt_) were inserted to simulate OSC devices. These values are consistent with optimized device conditions reported in literature and reflect efficient charge extraction and minimal leakage current. The designed SA1 compound with an ionization energy (IP) of 5.6 eV is used as a novel electron donor material. A typical [6,6]-phenyl-C71-butyric acid methyl ester (PC_71_BM) is utilized as an acceptor layer. The OSC components demonstrated in Fig. 2a were employed in the development of the model, including an indium tin oxide (ITO) and platinum (Pt) metal as front and back electrodes, respectively. The work function values of the ITO and Pt contacts were assigned to be 4 eV and 5.4 eV, respectively.^33,34^Tables 1 and 2 display the essential characteristics of the primary OSC (ITO/PC_71_BM/SA1/Pt) obtained by integrating the available experimental and theoretical results.^35–41^ The defect density at the SA1/PC_71_BM interface was 10^11^ cm^−2^, while the carrier charge's thermal velocity (*V*_th_) was maintained at 10^7^ cm s^−1^. As shown in Fig. 2b, the ultraviolet-visible (UV-vis) spectrum of SA1 has a broad absorption range from 350 nm to 750 nm, with a pronounced peak close to 600 nm, indicating that SA1 could effectively absorb photons in the visible range. This high absorption in the visible region is further supported by the EQE of the OSC device (Fig. 2f). The absorption is ascribed to π–π electronic transitions enabled by the extensive coupling of the dibenzo[*a*,*c*]phenazine and thieno[3,2-*b*]thiophene moieties.

**Table 1:** Description of parameters utilized in the planar heterojunction OSCs

Parameters	SA1	PC_71_BM	Y6-4O	ITIC-4F	o-BTP-eC9
Thickness (nm)	300	400	500	500	500
*E*_g_ (eV)	1.848	1.9	1.63	1.71	1.84
*χ*_e_ (eV)	3.2	3.92	4.01	4.02	3.86
HOMO (eV)	−5.677	−6.1	−5.64	−5.73	−5.70
LUMO (eV)	−3.829	−4.2	−4.01	−4.02	−3.86
*ε*_r_	8	6	5.13	3.94	2.9
*N*_C_ (cm^−3^)	1.0 × 10^20^	1.0 × 10^19^	1.0 × 10^19^	1.0 × 10^19^	1.0 × 10^19^
*N*_V_ (cm^−3^)	1.0 × 10^19^	1.0 × 10^20^	1.0 × 10^20^	1.0 × 10^20^	1.0 × 10^20^
*μ*_e_ (cm^2^ V^−1^ s^−1^)	5 × 10^−2^	1 × 10^−2^	0.92 × 10^−4^	2.93 × 10^−4^	3.5 × 10^−4^
*μ*_h_ (cm^2^ V^−1^ s^−1^)	5 × 10^−2^	1 × 10^−2^	0.92 × 10^−4^	2.93 × 10^−4^	4 × 10^−4^
*N*_D_ (cm^−3^)	1.0 × 10^15^	—	—	—	—
*N*_A_ (cm^−3^)	—	1.0 × 10^18^	1.0 × 10^18^	1.0 × 10^18^	1.0 × 10^18^
*N*_T_ (cm^−3^)	1.0 × 10^15^	1.0 × 10^14^	1.0 × 10^14^	1.0 × 10^14^	1.0 × 10^14^

**Table 2:** Interface properties of ITO/PC_71_BM/SA1/Pt planar OSCs

Parameters/interfaces	SA1/PC_71_BM
Type of defect	Neutral
Cross-section for electron capture (cm^2^)	1.0 × 10^−19^
Cross-section for hole capture (cm^2^)	1.0 × 10^−19^
Distribution of energies	Single
Reference for energy level of defect (*E*_t_)	Above the highest *E*_v_
Energy measured with respect to reference (eV)	0.600
Defect density (cm^−2^)	1.0 × 10^11^

Fig. 2c displays the energy band diagram after contact. The energy level scheme indicates properly matched energy levels that promote effective carrier separation and transportation. The conduction band and valence band demonstrate band bending, facilitating the formation of a built-in potential (*V*_bi_) at the interface, which promotes exciton dissociation and decreases the recombination rate (Fig. 2d). The obtained *J*–*V* photovoltaic characteristic of the primary OSC is shown in Fig. 2e. From our initial calculations, the designed OSC revealed an efficiency of 6.02%, a FF of 35.23%, a *V*_OC_ of 1.109 V, and a *J*_SC_ of 15.408 mA cm^−2^. Additionally, the EQE of the designed OSC is exhibited in Fig. 2f. The SA1 material in this OSC device exhibits efficient light-harvesting capability within the wavelength range of 300–600 nm. Fig. 2g illustrates that a significant generation rate is attained for the OSC device, which correlates with elevated *J*_SC_ and PCE values.

## 3. Results and discussion

**Fig. 3:**
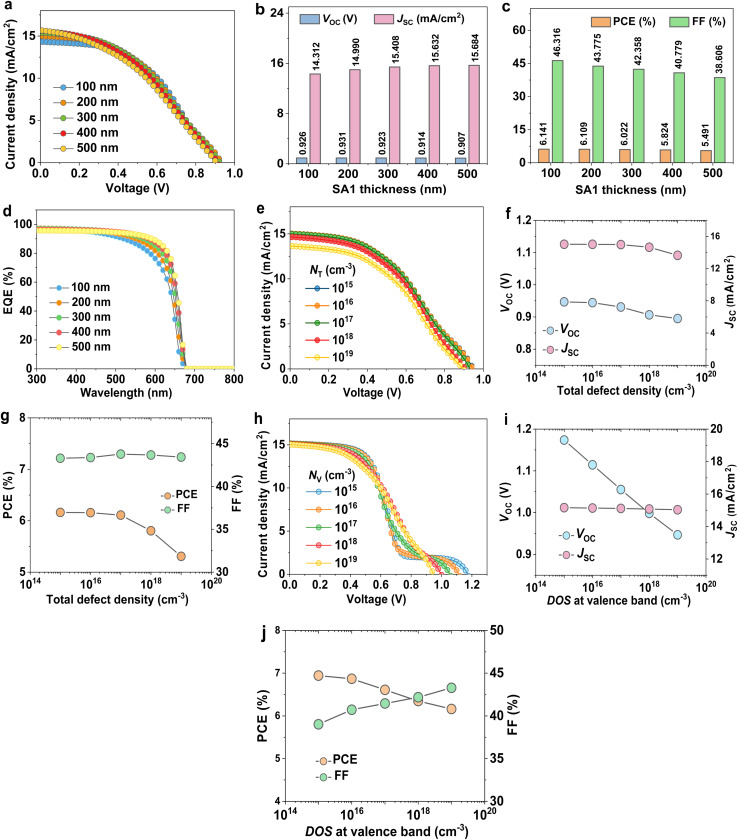
Optimization of the designed SA1 organic compound. (a) *J*–*V* curves of cells at various thicknesses. The related cell parameters: (b) *V*_OC_, *J*_SC_, (c) FF, PCE, and (d) EQE. (e) The variation defect density on *J*–*V* plots. Variation in (f) *V*_OC_, *J*_SC_, (g) FF, and PCE. (h) *J*–*V* plots variation with DOS at the valence band. Related parameters are (i) *V*_OC_, *J*_SC_, (j) FF, and PCE.

To optimize the DFT-designed SA1 compound, we probe the impact of SA1 thickness variation on photovoltaic parameters. At first, it is obvious from Fig. 3a that SA1-based OSC is recording the optimal performance at 200 nm, *V*_OC_ = 0.931 V, *J*_SC_ = 14.99 mA cm^−2^, FF = 43.775%, and PCE = 6.109%. As shown in Fig. 3b, the *J*_SC_ demonstrated a marginal improvement with increased SA1 thickness and eventually reached a saturation point at a 400 nm-thick layer. The improvement in *J*_SC_ can originate from improved absorption of long-wavelength photons, which raises the generation of electron–hole pairs. In cases where the photoactive thickness is low, only a limited number of long-wavelength photons are captured, resulting in a low production of carriers and, consequently, a reduced *J*_SC_.^42^ These results are consistent with the EQE spectral response (Fig. 3d). The highest EQE response of the OSC based on SA1 was reached at 400–500 nm. Generally, the FF of OSCs reduces with the increase of absorber thickness (see Fig. 3c). The FF of OSCs is reduced severely from 46.316% to 38.606% when the thickness increases from 100 nm to 500 nm.^43^ Actually, due to their short lifetime and prominent low exciton diffusion length (*L*), traditional OSC optimum performance is usually attained with a relatively thin photoactive layer, not more than 150 nm.^44^

The total defect density (*N*_T_) value in the SA1 active film changed within the range of 10^15^ to 10^19^ cm^−3^ to investigate the most favorable defect density for the designed OSCs. Fig. 3e–g illustrate the relationship between the *N*_T_ variation and the OSC output parameters, implying a notable reduction in PCE and *V*_OC_ as the *N*_T_ increases. The increase in recombination rate reduces the *L* value, thereby decreasing the efficiency. Prajapati and co-workers have adopted a similar effect.^45^ The desirable *N*_T_, achieving a PCE of around 6.109% for this specific cell, was determined to be 10^16^ cm^−3^.

Fig. 3h shows *J*–*V* characteristic curves of OSCs with various densities of states at the valence band (*N*_V_) of the SA1 compound. The *V*_OC_ can be given by:6
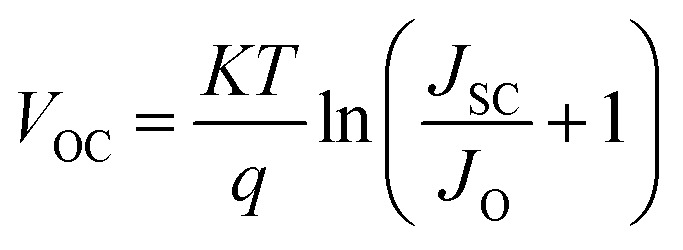
where *J*_o_ is the reverse saturation current density given by:7
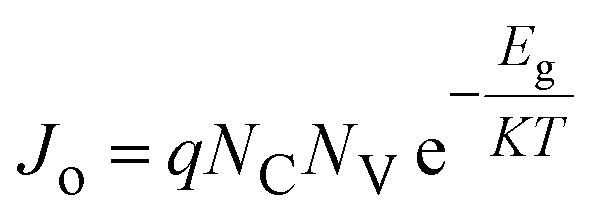
when *N*_V_ increases, *J*_o_ also increases, leading to a lower *V*_OC_, as depicted in Fig. 3i. A higher *N*_V_ indicates that more available states in the valence band lead to increased recombination processes, further suppressing *V*_OC_. Since efficiency is directly related to *V*_OC_, a decline in *V*_OC_ decreases the overall performance (see Fig. 3j).

**Fig. 4:**
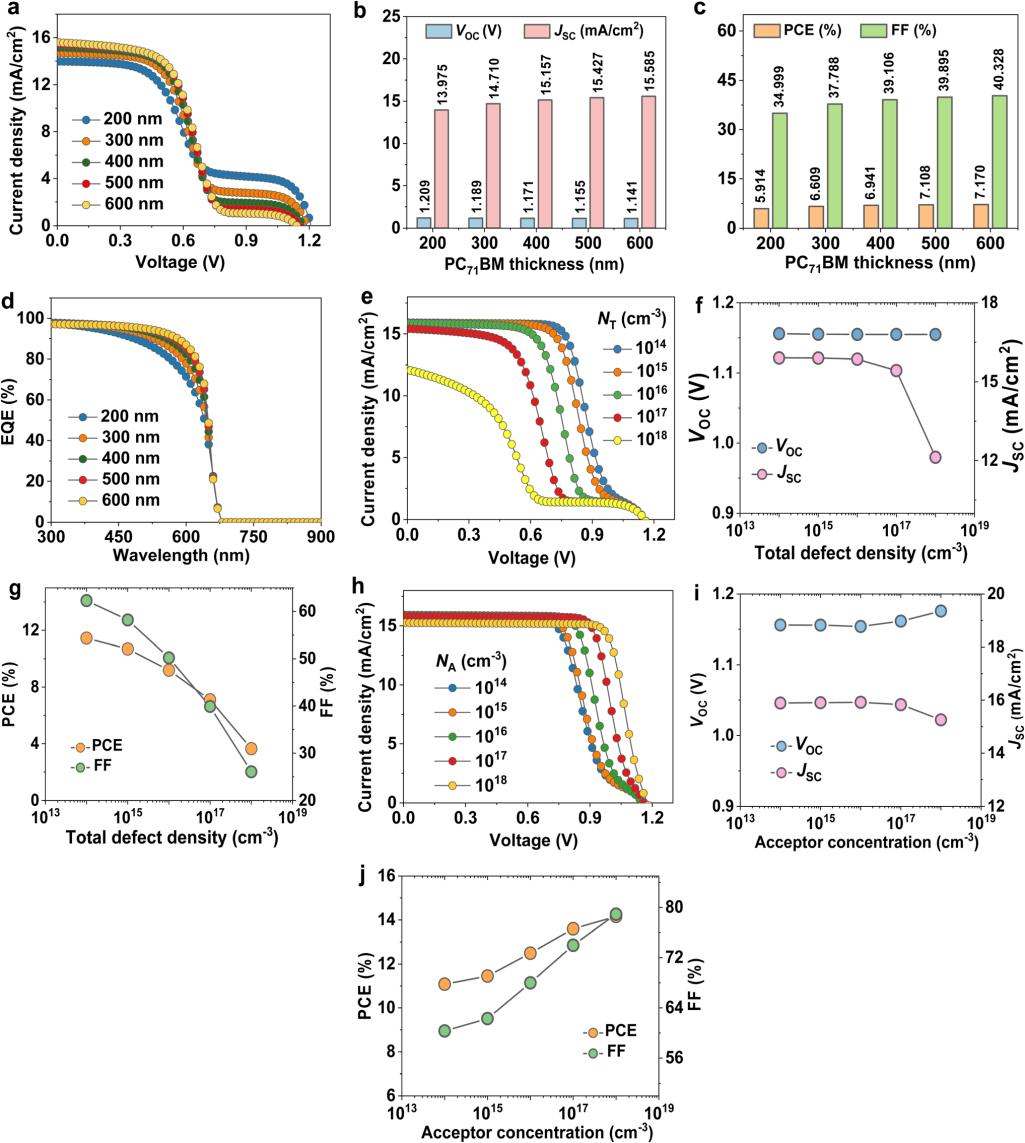
Optimization of the PC_71_BM acceptor compound. (a) *J*–*V* profiles of OSCs with different thicknesses. The related cell parameters: (b) *V*_OC_, *J*_SC_, (c) FF, PCE, and (d) EQE. (e) The variation bulk defect density on *J*–*V* plots. Variation in (f) *V*_OC_, *J*_SC_, (g) FF, and PCE. (h) *J*–*V* curves at different shallow acceptor concentration (*N*_A_). Corresponding parameters are (i) *V*_OC_, *J*_SC_, (j) FF, and PCE.

To further improve the photovoltaic properties of SA1-based OSC, the PC_71_BM acceptor layer was optimized. Regarding the optimization process, the thickness of the PC_71_BM film is altered to investigate the influence of its variation on the device performance. The thickness of PC_71_BM is changed from 200 nm to 600 nm, as shown in Fig. 4a. As the thickness increases, the *J*_SC_ increases, as depicted in Fig. 4b, due to increasing light absorption and photo-induced electron–hole pairs. As shown in Fig. 4c, the PC_71_BM thickness of 500 nm was chosen as it provides an optimized PCE by balancing efficiency and voltage.^46^ Additionally, a remarkable enhancement in FF was shown with increasing PC_71_BM thickness, which is related to suppressing *R*_S_ and improving charge transportation. The optimized PC_71_BM film promotes effective electron collection while mitigating recombination losses at the donor–acceptor interface (see Fig. 4d).

Furthermore, we explore the impact of the variation of bulk trap density on the photovoltaic merits of OSC devices. The calculation findings of the variation of the defect states from 10^14^ cm^−3^ to 10^18^ cm^−3^ on the *J*–*V* plot are demonstrated in Fig. 4e. As can be noticed from Fig. 4f and g, PC_71_BM defect density has a significant effect on the functionality of the OSCs. As defect concentration increases, decomposition in device parameters occurs because of increasing traps and recombination sites. Increasing defects leads to a reduction in the charge lifetime, and thus the recombination process increases. At 10^14^ cm^−3^ defect concentration, the OSC device achieved optimum performance with a *V*_OC_ of 1.155 V, a *J*_SC_ of 15.904 mA cm^−2^, a FF of 62.297%, and a PCE of 11.452%.

Dopant attempts to facilitate effective charge transfer, hence enhancing conductivity and balancing energy bands with the electrodes and the absorber interface, which results in increased efficiency. To characterize doping influence on device parameters, we changed the acceptor doping concentration (*N*_A_) of PC_71_BM from 10^14^ cm^−3^ to 10^18^ cm^−3^ (Fig. 4h). As depicted in Fig. 4i, a slight reduction in *J*_SC_ value was observed beyond the 10^17^ cm^−3^ doping level. Therefore, optimizing the doping concentration within the moderate range can help maintain efficient charge extraction and minimize recombination losses. As the *N*_A_ increased, a remarkable enhancement in FF and efficiency was recorded due to the *V*_bi_ effect.^47^ This improvement in the *V*_bi_ improves the extraction of carriers, giving rise to FF, as shown in Fig. 4j. Considering all these factors, the best magnitude for the *N*_A_ of PC_71_BM was selected to be 10^18^ cm^−3^. The device outputs at this concentration are PCE = 14.157%, FF = 78.916%, *V*_OC_ = 1.175 V, and *J*_SC_ = 15.257 mA cm^−2^.

**Fig. 5:**
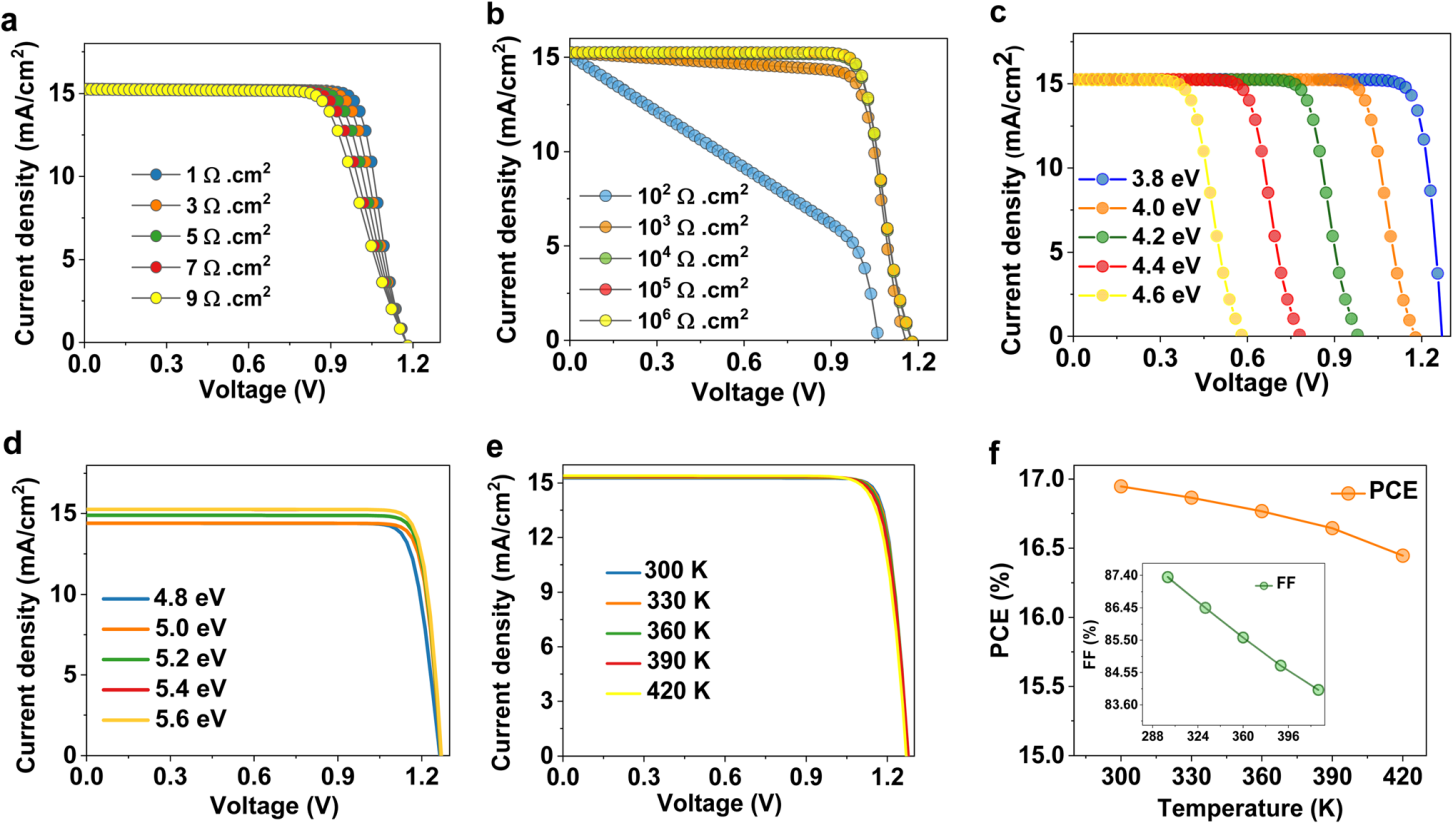
Optimization of parasitic resistances, electrodes, and operational temperature. (a) *J*–*V* plots of OSCs at different *R*_S_. (b) *J*–*V* plots of OSCs at different *R*_Shunt_. (c) *J*–*V* characteristics of OSCs at different work functions of front electrode. (d) *J*–*V* profiles of cells at different work functions of back electrode. (e) *J*–*V* curves and (f) device parameters at different temperatures.

The influence of *R*_S_ on the photovoltaic efficiency of the OSC was thoroughly analyzed by altering *R*_S_ from 1 Ω cm^2^ to 9 Ω cm^2^, as shown in Fig. 5a. The findings indicated a notable reduction in FF and PCE, although *V*_OC_ and *J*_SC_ remained unchanged (Table 3). This tendency is ascribed to increased ohmic losses, which impede carrier collection and induce extra voltage drops within the cell, hence diminishing the maximum energy output.^48^ The reduction in FF immediately affects PCE, emphasizing the necessity of reducing *R*_S_ for effective carrier transfer. We simulated *R*_Shunt_ values between 10^2^ Ω cm^2^ and 10^6^ Ω cm^2^ to investigate how OSC efficiency is affected. Fig. 5b displays the *J*–*V* profiles varying with *R*_Shunt_. With rising *R*_Shunt_, the *V*_OC_, FF, and PCE all progressively improve; the *J*_SC_ stays constant. The *R*_Shunt_ value was raised, and the efficiency improved from 5.76% to 14.46%. The *V*_OC_ decreases severely at low *R*_Shunt_ due to increased leakage currents, while it remains nearly constant at higher *R*_Shunt_ values where leakage becomes negligible.^49^ Our calculation indicates that an efficiency of 14.46% can be obtained with an *R*_Shunt_ of 10^5^ Ω cm^2^ (Table 3).

**Table 3:** The variation of key parameters of OSCs with *R*_S_ and *R*_Shunt_

Resistance		*V*_OC_ (V)	*J*_SC_ (mA cm^−2^)	FF (%)	PCE (%)
Series resistance (Ω cm^2^)	1	1.175	15.259	80.123	14.375
	3	1.175	15.255	77.724	13.942
	5	1.175	15.252	75.333	13.510
	7	1.175	15.249	72.951	13.080
	9	1.175	15.246	70.576	12.652
Shunt resistance (Ω cm^2^)	10^2^	1.063	15.109	35.856	5.760
	10^3^	1.155	15.245	76.733	13.522
	10^4^	1.175	15.259	80.123	14.375
	10^5^	1.177	15.260	80.445	14.461
	10^6^	1.178	15.260	80.477	14.469

Fig. 5c reveals the variations in the metal work function of the right contact from 3.8 eV to 4.6 eV in relation to the photovoltaic outputs of the cells. As illustrated in Table 4, while the *J*_SC_ was comparable, the *V*_OC_, FF, and PCE showed a substantial drop. This decrease is caused by an increase in the carrier transporting barrier at the interface between the electrode and PC_71_BM, which in turn causes higher charge aggregation and recombination losses as a result of energy band misalignment.^50^

**Table 4:** The variation of key parameters of OSCs with front and back work functions

Work function of electrode (eV)		*V*_OC_ (V)	*J*_SC_ (mA cm^−2^)	FF (%)	PCE (%)
Front electrode	3.8	1.271	15.261	87.347	16.947
	4.0	1.178	15.260	80.477	14.469
	4.2	0.981	15.259	76.879	11.514
	4.4	0.781	15.258	71.987	8.583
	4.6	0.581	15.256	64.265	5.701
Back electrode	4.8	1.264	14.399	85.570	15.586
	5.0	1.269	14.403	87.430	15.980
	5.2	1.270	14.894	87.386	16.534
	5.4	1.271	15.261	87.347	16.947
	5.6	1.271	15.262	87.347	16.948

The back Pt metal operates as the hole-collecting electrode, requiring its work function to be compatible with the HOMO of the SA1 layer. The energy barrier of 4.8 eV between the HOMO of SA1 and Pt is considerable, resulting in diminished hole injection and charge accumulating at the interface (Fig. 5d). As the work function rises to 5.4 eV, the band matching enhances, leading to less interfacial charge accumulation and minimized recombination rates. This results in a marginal enhancement in *J*_SC_ as an increased number of photocarriers participate in the photocurrent. The optimized SA1-based OSC recorded a PCE of 16.94% at front and back work functions of 3.8 eV and 5.4 eV, respectively. The SA1-based OSC device achieved a high FF of 87.34%, reflecting effective charge-carrier transport and reduced recombination rates under the simulated conditions. However, the overall efficiency (16.94%) remains moderate due to limitations in photocurrent generation (15.26 mA cm^−2^), which are governed by the intrinsic absorption range of OSC.

The operational temperature of a solar cell influences its efficiency. Increasing the ambient temperature adversely affects the charge carrier mobility, resulting in reduced device performance.^51^ The operating temperature is assessed to be 300–420 K, and the measuring parameters are depicted in Fig. 5e and f. Figures indicate that the increment in temperature leads to a slight reduction in the *V*_OC_. The *V*_OC_ decline due to higher temperature leads to a shrinkage in the *E*_g_ of the light-harvesting material.^52^ Meanwhile, the *J*_SC_ parameter is unchanged due to increasing temperatures. Additionally, increasing temperatures induce more lattice vibrations, diminishing photo-induced carrier mobility because of intensified phonon scattering, which subsequently elevates *R*_S_ and decreases the FF. Higher thermal energy also promotes recombination rates, mainly SRH and Auger recombination processes, further restricting charge collection. Overall, there is a slight decrease in PCE from 16.94% to 16.06%. Thus, the simulated OSC with the SA1 compound is highly stable at higher temperatures.

**Fig. 6:**
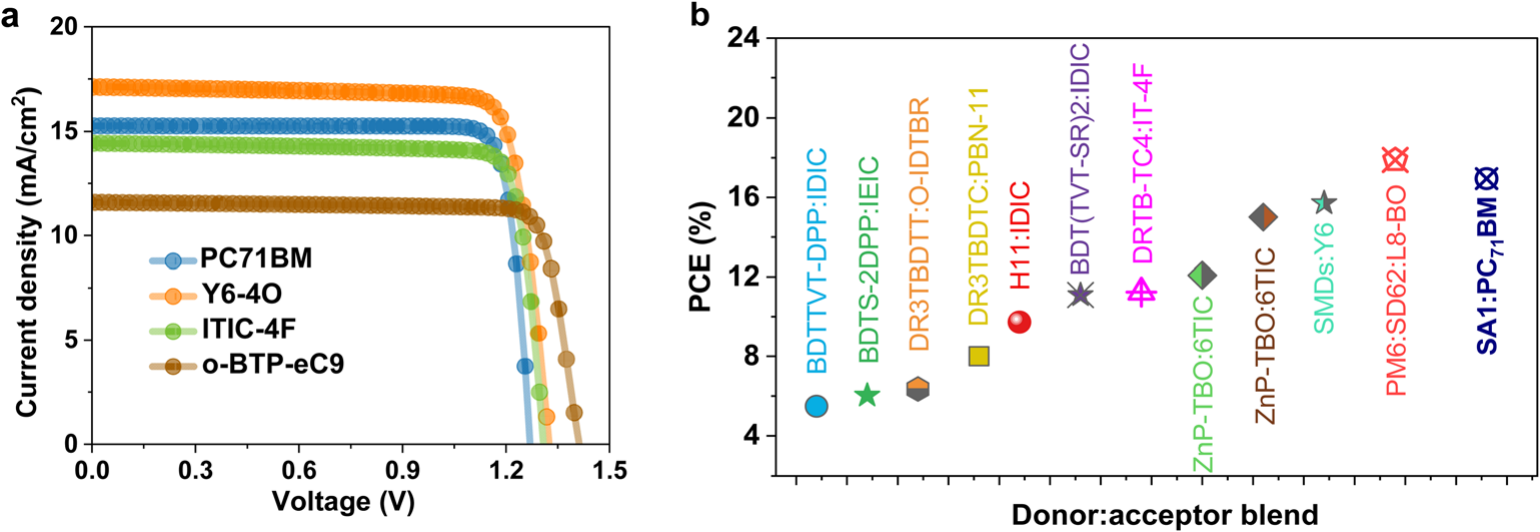
The final optimized SA1-based OSC and comparison with different non-fullerene acceptor devices. (a) *J*–*V* characteristics of simulated OSCs based on different acceptors. (b) Comparison of SA1-OSC with literature-reported SM donors.

To evaluate the versatility and practical applicability of the newly designed SM donor SA1, we simulated its combination with four different acceptors: the traditional fullerene-based PC_71_BM and three state-of-the-art non-fullerene acceptors (NFAs)—Y6, ITIC-4F, and BTP-eC9. As shown in Fig. 6a, all OSCs share an identical architecture, differing only in the acceptor layer, which enables a direct assessment of the acceptor's influence on device performance. Notably, the SA1:Y6-4O cell showed the best-performing PCE, driven by high *J*_SC_ and FF. This pattern can be attributed to Y6-4O's broad absorption spectrum extending into the near-infrared region, its favorable energy level alignment with SA1, and its high dielectric constant, which together facilitate effective exciton dissociation and charge carrier collection. The cells based on ITIC-4F and o-BTP-eC9 showed high photovoltaic performance, with efficiencies that closely rival that of Y6-4O. Table 5 and Fig. 6b summarize a comparison of our proposed SA1-based OSC with existing reported SM donor structures. With an ITO/SA1/PC_71_BM/Pt pattern, the cell developed in this study had a significantly high PCE of 16.94% and a FF of 87.34%, exceeding several previously published SM-based OSCs. Regarding thermal stability, our device maintained 94% of its starting efficiency under 145 °C, surpassing previous systems displaying performance loss under comparable or less harsh environments.

**Table 5:** Description of photovoltaic characteristics of reported small molecule-based organic solar cells^a^

Author	Structure	*V*_OC_ (V)	*J*_SC_ (mA cm^−2^)	FF (%)	PCE (%)	Stability
Huo *et al.*^53^	ITO/PEDOT:PSS/BDTTVT-DPP:IDIC/PDIN/Al	0.82	11.30	59.17	5.48	Not reported
Lin *et al.*^54^	ITO/ZnO/BDTS-2DPP:IEIC/MoO_*x*_/Ag	0.94	10.87	59.00	6.03	Not reported
Yang *et al.*^55^	ITO/PEDOT:PSS/DR3TBDTT:O-IDTBR/BCP/Al	1.15	11.06	50.00	6.36	Not reported
Zhang *et al.*^56^	TO/ZnO/DR3TBDTC:PBN-11/MoO_3_/Al	1.11	11.18	64.60	8.01	Retain 89% after 168 hours at 180 °C and 74% after 72 hours under illumination
Bin *et al.*^57^	ITO/PEDOT:PSS/H11:IDIC/PDINO/Al	0.97	15.21	65.46	9.73	Not reported
Guo *et al.*^58^	ITO/PEDOT:PSS/BDT(TVT-SR)_2_:IDIC/PDINO/Al	0.98	15.92	71.15	11.10	Retain 49% after 96 hours at 80 °C and 90% after 200 hours under illumination
Yang *et al.*^59^	ITO/ZnO/DRTB-TC4:IT-4F/MoO_3_/Al	0.91	18.27	68.00	11.24	Not reported
Gao *et al.*^60^	ITO/PEDOT:PSS/ZnP-TBO:6TIC/C_60_-bissalt/Ag	0.80	20.44	73.87	12.08	Retain 97% after 100 hours in air (25 °C, humidity <15%)
Gao *et al.*^61^	ITO/PEDOT:PSS/SMD:BO-4Cl/PFN-Br:MA/Ag	0.84	24.38	72.7	15.00	Retain 70% after 450 hours at 80 °C
Guo *et al.*^62^	ITO/PEDOT:PSS/SMDs:Y6/PDINN/Ag	0.84	26.64	69.70	15.71	Retain 75% after 225 hours under illumination
Wang *et al.*^63^	ITO/PEDOT:PSS/PM6:SD62:L8-BO/PNDIT F3N/Ag	0.88	26.08	77.75	17.88	Retain 75% after 250 hours under illumination
**This work**	**ITO/SA1/PC**_**71**_ **BM/Pt**	**1.27**	**15.26**	**87.34**	**16.94**	Retain 94% at 145 °C

a PEDOT-PSS: poly(3,4-ethylenedioxythiophene)-poly-(styrene-sulfonate); PDINO: perylene diimide functionalized with amino *N*-oxide.

**Fig. 7:**
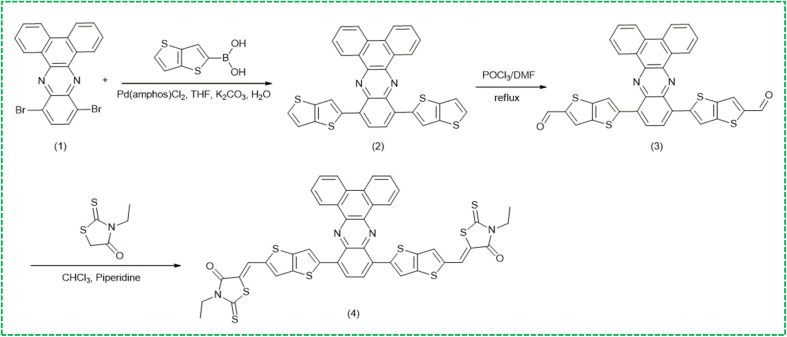
The synthetic pathway suggestion of SA1 compound.

Finally, to ensure the designed SA1 compound is not only theoretically promising but also synthetically viable, a three-step synthetic route is suggested, inspired by established organic methodologies commonly applied in the synthesis of π-conjugated donor materials. The synthesis of SA1 could involve three reaction steps (Fig. 7). First, a Suzuki–Miyaura coupling reaction occurs between 10,13-dibromodibenzo[*a*,*c*]phenazine (1) and thieno[3,2-*b*]thiophen-2-ylboronic acid, leading to the formation of compound (2). Next, compound (3) is synthesized *via* the Vilsmeier–Haack reaction. Lastly, the resulting compound undergoes a Knoevenagel condensation reaction to yield SA1.

## 4. Conclusion

In summary, a novel phenazine-based SM compound, SA1, was computationally designed for planar-heterojunction OSCs. We explored the potential of SA as the electron donor layer in SM-based OSCs. The developed ITO/PC_71_BM/SA1/Pt architecture was systematically optimized for thickness, bulk defect states, doping level, and effective density of states concentration using SCAPS-1D numerical software. The findings indicate that the preferred thicknesses of SA1 and PC_71_BM are 200 nm and 500 nm, respectively. Besides, optimizing the PC_71_BM doping level to 10^18^ cm^−3^ was determined to be beneficial for increasing the simulated OSC's performance. Similarly, the optimal defect levels are measured as 10^16^ cm^−3^, and 10^14^ cm^−3^ for the SA1 and PC_71_BM, respectively. Our results reveal that by suppressing *R*_S_ and increasing *R*_Shunt_, the cell performance can be improved. The optimized OSC device showed remarkable performance, obtaining a PCE of 16.94%, FF of 87.34%, a *V*_OC_ of 1.27 V, a *J*_SC_ of 15.26 mA cm^−2^, and an EQE of 95%. The temperature dependencies of photovoltaic parameters were examined across the range of 300 K to 420 K to assess heating stressors. Interestingly, the designed OSC structure showed stable behavior over this temperature range, suggesting good predicted thermal tolerance. The comparison of PC_71_BM-based OSCs with three different NFAs (Y6-4O, ITIC-4F, and o-BTP-eC9) exhibited that the acceptor layer influences device performance. Among them, the SA1:Y6-4O device showed the most promising photovoltaic parameters, highlighting the synergy between the designed donor and state-of-the-art acceptors.

## Conflicts of interest

The authors declare no conflict of interest.

## Data Availability

The data will be available from the corresponding author on reasonable request.
